# Diagnostic Timing and Ovarian Cancer Survival

**DOI:** 10.1001/jamanetworkopen.2026.2434

**Published:** 2026-03-27

**Authors:** Sarah E. Soppe, Tzy-Mey Kuo, Victoria L. Bae-Jump, Benjamin B. Albright, Georgios Lyratzopoulos, Chris D. Baggett, Caroline A. Thompson

**Affiliations:** 1 Department of Epidemiology, Gillings School of Global Public Health, University of North Carolina at Chapel Hill, Chapel Hill; 2 Lineberger Comprehensive Cancer Center, University of North Carolina at Chapel Hill, Chapel Hill; 3 Division of Gynecologic Oncology, University of North Carolina at Chapel Hill, Chapel Hill; 4 Epidemiology of Cancer Healthcare and Outcomes, Department of Behavioural Science and Health, Institute of Epidemiology and Health Care, University College London, London, United Kingdom

## Abstract

**Importance:**

While earlier diagnosis improves outcomes for many cancers, studies report that earlier diagnosis does not improve ovarian cancer (OC) survival, discouraging investment in diagnostic improvements. One possible explanation is confounding due to the wait time paradox, in which patients with severe disease are diagnosed more quickly but have poorer outcomes, masking potential benefits of earlier diagnosis.

**Objective:**

To visualize how nonlinear modeling of time to diagnosis alters observed associations with OC survival, with particular attention to the wait time paradox.

**Design, Setting, and Participants:**

This cohort study used data from the North Carolina Central Cancer Registry with linked multipayer claims from Medicare, Medicaid, and private insurance. Participants included female patients diagnosed with OC from January 1, 2009, to December 31, 2019, in North Carolina. Follow-up was completed June 30, 2021. Data were analyzed from May 1, 2024, to November 1, 2025.

**Exposure:**

Time to diagnosis, defined as the interval from the earliest symptom-related health care encounter to cancer diagnosis.

**Main Outcomes and Measures:**

All-cause 5-year mortality.

**Results:**

Among 2359 women included in the analysis (median age, 71 [IQR, 64-78] years), the median diagnostic interval was 33 (IQR, 10-149) days. Compared with all other quartiles, patients in the second-shortest quartile of the diagnostic interval were more likely to be diagnosed at localized (83 of 586 [14.2%] vs 180 of 1773 [10.2%]) or regional (134 of 586 [22.9%] vs 330 of 1773 [18.6%]) stages (*P* = .001) and had a median survival exceeding 5 years (lower 95% CI bound, 4.1 years), compared with median survivals of 2.5 years (95% CI, 2.0-3.1 years) in the shortest quartile and 2.9 years (95% CI, 2.5-3.8 years) in the longest quartile. When modeled using restricted cubic splines, the adjusted association between time to diagnosis and mortality was U-shaped; compared with a reference interval of 80 days, intervals of 10 and 360 days were associated with higher mortality (hazard ratios, 1.29 [95% CI, 1.09-1.53] and 1.13 [95% CI, 0.88-1.46], respectively).

**Conclusions and Relevance:**

In this cohort study of patients with OC, very short diagnostic intervals were associated with poorer survival, consistent with residual confounding by the wait time paradox, while longer diagnostic intervals were also associated with worse outcomes. Conventional linear approaches may obscure this important confounding by severity in the association between diagnostic timing and survival, contributing to null findings in prior studies and potentially discouraging efforts to improve diagnosis of this difficult-to-detect disease.

## Key Points

**Question:**

Does the wait time paradox (in which patients with severe disease are diagnosed quickly but have poor prognosis) contribute to observed associations between diagnostic timing and survival of ovarian cancer?

**Findings:**

In this cohort study using registry-linked claims data from 2359 patients with ovarian cancer, flexible nonlinear modeling demonstrated a U-shaped association between diagnostic timing and mortality, with poorer survival at very short and very long diagnostic intervals compared with intermediate intervals, patterns consistent with confounding by the wait time paradox.

**Meaning:**

These findings suggest that conventional linear modeling approaches may obscure the impact of the wait time paradox, potentially masking any benefit of earlier diagnosis.

## Teaser

This cohort study assesses whether nonlinear modeling of time to diagnosis alters observed associations with ovarian cancer survival, with particular attention to the wait time paradox.

## Introduction

Ovarian cancer (OC) is difficult to diagnose, as most patients present with nonspecific symptoms common in the general population.^1,2,3,4,5^ While national cancer registry estimates indicate the overall relative 5-year survival is 52%, patients diagnosed with localized disease have substantially longer survival (92%).^6^ This suggests that diagnosis earlier in the disease course may improve outcomes, as commonly assumed in cancer research.^7^ However, studies examining associations between OC outcomes and the diagnostic interval, or the time from the earliest symptom-related health care presentation to diagnosis, reported that shorter intervals were not associated with improved survival or stage.^8,9^ These findings have contributed to skepticism about investing in diagnostic improvement initiatives. Some have hypothesized that OC is too advanced by the time patients seek care for nonalarming symptoms, implying that the health care system cannot improve outcomes through earlier diagnosis.^10^ However, this contradicts evidence that patients may spend up to a year seeking diagnostic care before diagnosis, often receiving gastrointestinal diagnoses before OC is suspected.^1,2,3,4,5,11,12^ To justify investment in clinical tools to improve OC diagnosis, it is essential to clarify whether earlier diagnosis is associated with improved survival.

A recent systematic review^8^ suggested studies of the diagnostic interval and OC outcomes are affected by the wait time paradox, in which patients with more severe symptoms are diagnosed more quickly but have poorer survival, making shorter intervals appear associated with worse outcomes.^13^ In essence, the sickest patients are diagnosed fastest, creating an apparent inverse association between diagnostic speed and survival that obscures any true benefit of earlier diagnosis.^13^ This form of confounding by severity can be examined using nonlinear analytic approaches that allow diagnostic intervals of different lengths to have different associations with outcomes.^14^ Such methods have been applied in studies of other aggressive cancers,^14,15,16^ but remain underused for OC.

We examined diagnostic intervals and survival among patients with OC in North Carolina using nonlinear methods to visualize confounding by severity. Our aim was to determine whether evidence discouraging investment in diagnostic improvement reflects methodologic limitations rather than the true biological impact of earlier diagnosis.

## Methods

This cohort study was approved by the University of North Carolina (UNC) at Chapel Hill Institutional Review Board with a waiver of informed consent for use of deidentified data. The study followed the Strengthening the Reporting of Observational Studies in Epidemiology (STROBE) reporting guideline.

### Study Population

We used the UNC Cancer Information and Population Health Resource, which links data from the North Carolina Central Cancer Registry (NCCCR) to Medicare, Medicaid, and private insurance claims.^17^ We included women 18 years or older diagnosed symptomatically with epithelial OC from January 1, 2009, to December 31, 2019. Patients diagnosed at autopsy or without at least 12 months of continuous health care coverage in the year before diagnosis were excluded. Additional exclusions included lack of a reasonable index date to define diagnostic timing (n = 115), an earliest claim for OC symptoms on the date of surgery (n = 84), and no symptom-related claims on or before the index date (n = 146), yielding a final sample of 2359 patients.

### Diagnostic Interval

The diagnostic interval was defined as the number of days between the earliest symptom-related health care encounter in any clinical setting, prospectively captured in claims, and the index date marking clinical diagnosis. The index date was defined as the earliest of the following: an OC code from the *International Classification of Diseases, Ninth Revision* (183.0), and *International Statistical Classification of Diseases, Tenth Revision* (C56.1-C56.3 and C56.9) (collectively termed *ICD*), occurring within 1 month of the NCCCR diagnosis date,^18,19^ or the earliest surgical procedure associated with OC biopsy (eg, hysterectomy, laparoscopy, oophorectomy) occurring from the year before to 6 months after the NCCCR diagnosis date (eTable 1 in Supplement 1). The NCCCR date was not used to define the end of the interval because it does not reliably correspond to the date of clinical suspicion of OC.^19^

To identify the earliest presentation to health care, we used the earliest *ICD* code in claims indicating a symptom-related encounter on or before the index date within the prior year. These symptoms commonly used in clinical practice were grouped into categories: abdominal pain or tenderness, gastrointestinal symptoms, abdominal or pelvic swelling, and pelvic pain (specific codes in eTable 2 in Supplement 1).^20^ Multiple symptom categories were allowed for care occurring within 7 days of the earliest presentation. Because early OC symptoms are often attributed to more common conditions, claims for these diagnoses may reflect care for undiagnosed OC.^21^ To assess how many patients had visits for potentially related care not included in the main analysis, we conducted a sensitivity analysis defining the start date of the diagnostic interval using broader claims for symptomatically similar diseases (SSDs), including gastrointestinal and menopausal symptom diagnoses and urinary symptoms (eTable 3 and eFigure 1 in Supplement 1).^20^ The prevalence of these symptoms at the start of the interval are included in eTable 4 in Supplement 1.

### Statistical Analysis

Data were analyzed from May 1, 2024, to November 1, 2025. Patient characteristics were compared across quartiles of the diagnostic interval, including age, race and ethnicity, year of diagnosis, neighborhood-level socioeconomic status (SES),^22^ Surveillance, Epidemiology, and End Results summary stage, histologic subtype, epithelial type defined by the Kurman dualistic model,^23^ and insurance payer at the month of diagnosis. Race and ethnicity were abstracted from the medical record by the NCCCR and were categorized as Hispanic, non-Hispanic Black, non-Hispanic White, or another group or unknown; smaller groups including American Indian or Alaska Native, Asian American, Native Hawaiian or Pacific Islander, and multiracial were combined due to small sample sizes. Race and ethnicity data were included to compare demographic characteristics across the diagnostic interval quartiles, enabling assessment of potential disparities in diagnostic delay. Using claims from the year before diagnosis, we calculated the Charlson Comorbidity Index (CCI),^24^ excluding 6 months before diagnosis to minimize overlap with the diagnostic interval. Initial presentation to the emergency department (ED) was identified using ED revenue codes (0450-0459 and 0981). We calculated *P* values for differences in these variables by quartile of the diagnostic interval using χ^2^ tests; 2-sided *P* < .05 indicated statistical significance.

Dates of death were obtained from the NCCCR, and patients were censored at the earliest of 5 years after diagnosis or June 30, 2021. Unadjusted Kaplan-Meier curves were examined by diagnostic interval quartile. The nonlinear association between diagnostic interval length and survival was modeled using Cox proportional hazards regression models with restricted cubic splines, with knots at the percentiles recommended by Harrell.^25^ Models with 3 to 6 knots were evaluated, and the 5-knot model was selected based on the lowest Akaike information criterion. The proportional hazards assumption was assessed using Wald tests for time-varying interval terms. Crude hazard ratios (HRs) with 95% CIs were estimated for each 10-day interval from 0 to 360 days compared with the interval with the lowest hazard. We also calculated HRs adjusted for potential confounders identified a priori using directed acyclic graph methods,^26^ including age at diagnosis modeled using linear splines, CCI categories (0, 1, 2, or ≥3),^24^ quintiles of neighborhood-level SES,^22^ epithelial type based on the Kurman classification,^23^ categorical year of diagnosis, insurance payer (Medicare, Medicaid, or private), whether the initial presentation was to the ED, and the symptom categories at the earliest presentation modeled using binary indicator variables. To visualize how conclusions differ when the diagnostic interval is modeled linearly as in most prior studies, we also estimated HRs using a Cox proportional hazards regression model with a linear diagnostic interval term. As an additional sensitivity analysis, we adjusted for stage, a potential confounder and/or mediator of the interval and survival association.

## Results

**Figure 1.**
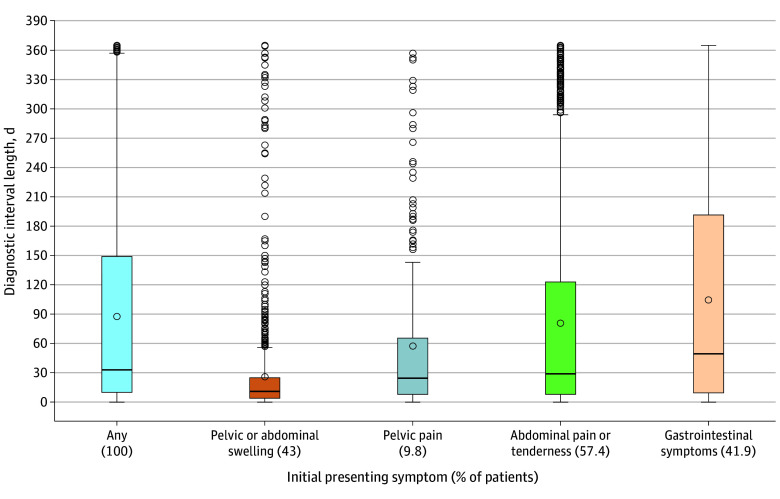
Box Plot Showing Diagnostic Interval Length by Initial Presenting Symptom to the Health Care System Circles indicate means; colored bars, IQRs; error bars, the smallest value within 1.5 times the IQR below quartile 1 and the largest value within 1.5 times the IQR above quartile 3; and horizontal lines, medians.

Among 2359 patients with symptomatic OC included in the analysis (median age, 71 [IQR, 64-78] years), the diagnostic interval had a median of 33 (IQR, 10-149) days and a mean (SD) of 88 (107) days (Figure 1). Intervals ranged from 0 to 365 days based on the available lookback window. Patients initially presenting with pelvic or abdominal swelling had the shortest mean (SD) intervals (26 [52] days), followed by pelvic pain (57 [79] days), abdominal pain or tenderness (81 [103] days), and gastrointestinal symptoms (105 [114] days). When including SSDs and urinary symptoms, the median interval length was 113 (IQR, 21-269) days with a mean (SD) of 145 (127) days. Intervals were substantially longer when defined by a gastrointestinal disorder or menopausal symptom diagnosis in the sensitivity analysis (mean [SD], 187 [125] days and 207 [112] days, respectively) (eTable 4 in Supplement 1).

In the entire cohort, 36 patients (1.5%) were Hispanic, 319 (13.5%) were non-Hispanic Black, 1953 (82.8%) were non-Hispanic White, and 51 (2.2%) were of another or unknown race. Most patients had distant staged disease (1550 [65.7%]) and a more aggressive Kurman epithelial type (type II; 1959 [83.0%]), with the overall cohort representing a broad range of ages and neighborhood-level SES (Table). Compared with all other quartiles, patients in the second shortest quartile were younger (141 of 586 [24.1%] <60 years old vs 281 of 1773 [15.8%]), were more likely to be non-Hispanic White (500 of 586 [85.3%] vs 1453 of 1773 [82.0%]), had higher neighborhood-level SES (260 of 586 [44.4%] in the top 2 SES quintiles vs 745 of 1773 [42.0%]), had fewer comorbidities (449 of 586 [76.6%] with CCI of 0 vs 1174 of 1773 [66.2%]), had localized (83 of 586 [14.2%] vs 180 of 1773 [10.2%]) or regional (134 of 586 [22.9%] vs 330 of 1773 [18.6%]) stage cancer, had less aggressive epithelial types (126 of 586 [21.5%] type I vs 274 of 1773 [15.5%]), and had lower probability of having presented to the ED (91 of 586 [15.5%] vs 394 of 1773 [22.2%]). In contrast, compared with all other quartiles, patients in the longest interval quartile had the lowest proportion of non-Hispanic White patients (470 of 590 [79.7%] vs 1483 of 1769 [83.8%]) and the most patients in the 2 most deprived quintiles of SES (237 of 590 [40.2%] vs 663 of 1769 [37.5%]). Some patients were diagnosed after only 1 visit for an included symptom (285 [12.1%]), with this proportion declining dramatically over the quartiles from 198 patients (34.1%) in the shortest quartile to 19 (3.2%) in the longest quartile. More than 40% of patients who initially presented to the ED were in the shortest interval quartile (200 of 485 [41.2%]). The shortest interval quartile also had the greatest proportion of patients with distant stage (412 of 581 [70.9%]), followed by the fourth quartile (397 of 590 [67.3%]), third quartile (388 of 602 [64.5%]), and second quartile (353 of 586 [60.2%]).

**Table.** Demographic and Clinical Characteristics of Patients With Ovarian Cancer by Quartile of the Diagnostic Interval Length

Characteristics	Overall, No. (%) (N = 2359)	Quartile of diagnostic interval length, No. (column %) [row %]				*P* value
		<10 d (n = 581)	10-32 d (n = 586)	33-148 d (n = 602)	149-365 d (n = 590)	
Age at diagnosis, y						
20-49	177 (7.5)	40 (6.9) [22.6]	53 (9.0) [29.9]	37 (6.1) [20.9]	47 (8.0) [26.6]	<.001
50-59	245 (10.4)	41 (7.1) [16.7]	88 (15.0) [35.9]	63 (10.5) [25.7]	53 (9.0) [21.6]	
60-69	588 (24.9)	170 (29.3) [28.9]	144 (24.6) [24.5]	154 (25.6) [26.2]	120 (20.3) [20.4]	
70-79	840 (35.6)	198 (34.1) [23.6]	196 (33.4) [23.3]	231 (38.4) [27.5]	215 (36.4) [25.6]	
>79	509 (21.6)	132 (22.7) [25.9]	105 (17.9) [20.6]	117 (19.4) [23.0]	155 (26.3) [30.5]	
Race and ethnicity						
Hispanic	36 (1.5)	<18 (<3.1) [<50.0]	<20 (<3.2) [<55.6]	<22 (<3.7) [<61.1]	13 (2.2) [<36.1]	.41
Non-Hispanic Black	319 (13.5)	76 (13.1) [23.8]	66 (11.3) [20.7]	84 (14.0) [26.3]	93 (15.8) [29.2]	
Non-Hispanic White	1953 (82.8)	487 (83.8) [24.9]	500 (85.3) [25.6]	496 (82.4) [25.4]	470 (79.7) [24.1]	
Other or unknown^a^	51 (2.2)	<18 (<3.1) [<35.3]	<20 (<3.2) [<39.2]	<22 (<3.7) [<43.1]	14 (2.4) [<27.5]	
Year of diagnosis						
2009-2010	442 (18.7)	119 (20.5) [26.9]	108 (18.4) [24.4]	98 (16.3) [22.2]	117 (19.8) [26.5]	.21
2011-2013	598 (25.3)	147 (25.3) [24.6]	167 (28.5) [27.9]	141 (23.4) [23.6]	143 (24.2) [23.9]	
2014-2016	646 (27.4)	154 (26.5) [23.8]	159 (27.1) [24.6]	167 (27.7) [25.9]	166 (28.1) [25.7]	
2017-2019	673 (28.5)	161 (27.7) [23.9]	152 (25.9) [22.6]	196 (32.6) [29.1]	164 (27.8) [24.4]	
Census-tract level SES quintile						
First (most deprived)	424 (18.0)	110 (18.9) [25.9]	100 (17.1) [23.6]	109 (18.1) [25.7]	105 (17.8) [24.8]	.69
Second	476 (20.2)	119 (20.5) [25.0]	107 (18.3) [22.5]	118 (19.6) [24.8]	132 (22.4) [27.7]	
Third	>443 (>18.8)	>100 (>17.2) [<100]	>108 (>18.4) [<100]	>111 (>18.4) [<100]	>91 (>15.4) [<100]	
Fourth	511 (21.7)	116 (20.0) [22.7]	145 (24.7) [28.4]	124 (20.6) [24.3]	126 (21.4) [24.7]	
Fifth (least deprived)	494 (20.9)	125 (21.5) [25.3]	115 (19.6) [23.3]	129 (21.4) [26.1]	125 (21.2) [25.3]	
Unknown	<11 (<0.5)	<11 (<1.9) [<100]	<11 (<1.9) [<100]	<11 (<1.8) [<100]	<11 (<1.9) [<100]	
CCI (6-12 mo before diagnosis)						
0	1623 (68.8)	422 (72.6) [26.0]	449 (76.6) [27.7]	411 (68.3) [25.3]	341 (57.8) [21.0]	<.001
1	419 (17.8)	94 (16.2) [22.4]	82 (14.0) [19.6]	112 (18.6) [26.7]	131 (22.2) [31.3]	
2	167 (7.1)	34 (5.9) [20.4]	37 (6.3) [22.2]	39 (6.5) [23.4]	57 (9.7) [34.1]	
≥3	150 (6.4)	31 (5.3) [20.7]	18 (3.1) [12.0]	40 (6.6) [26.7]	61 (10.3) [40.7]	
Insurance payer at month of diagnosis						
Private	400 (17.0)	94 (16.2) [23.5]	146 (24.9) [36.5]	88 (14.6) [22.0]	72 (12.2) [18.0]	<.001
Medicaid	425 (18.0)	113 (19.4) [26.6]	83 (14.2) [19.5]	102 (16.9) [24.0]	127 (21.5) [29.9]	
Medicare	1534 (65.0)	374 (64.4) [24.4]	357 (60.9) [23.3]	412 (68.4) [26.9]	391 (66.3) [25.5]	
SEER summary stage						
Localized	263 (11.1)	46 (7.9) [17.5]	83 (14.2) [31.6]	72 (12.0) [27.4]	62 (10.5) [23.6]	.001
Regional	464 (19.7)	99 (17.0) [21.3]	134 (22.9) [28.9]	127 (21.1) [27.4]	104 (17.6) [22.4]	
Distant	1550 (65.7)	412 (70.9) [26.6]	353 (60.2) [22.8]	388 (64.5) [25.0]	397 (67.3) [25.6]	
Unknown	82 (3.5)	24 (4.1) [29.3]	16 (2.7) [19.5]	15 (2.5) [18.3]	27 (4.6) [32.9]	
Histologic subtype						
Clear cell carcinoma	96 (4.1)	21 (3.6) [21.9]	34 (5.8) [35.4]	26 (4.3) [27.1]	15 (2.5) [15.6]	<.001
Endometroid carcinoma	139 (5.9)	26 (4.5) [18.7]	40 (6.8) [28.8]	35 (5.8) [25.2]	38 (6.4) [27.3]	
Mixed epithelial-stromal carcinoma	179 (7.6)	44 (7.6) [24.6]	49 (8.4) [27.4]	37 (6.1) [20.7]	49 (8.3) [27.4]	
Mucinous carcinoma	92 (3.9)	19 (3.3) [20.7]	28 (4.8) [30.4]	30 (5.0) [32.6]	15 (2.5) [16.3]	
Low-grade serous carcinoma	204 (8.6)	45 (7.7) [22.1]	39 (6.7) [19.1]	50 (8.3) [24.5]	70 (11.9) [34.3]	
High-grade serous carcinoma	1067 (45.2)	259 (44.6) [24.3]	270 (46.1) [25.3]	295 (49.0) [27.6]	243 (41.2) [22.8]	
Undifferentiated or other epithelial type	>571 (>24.2)	>156 (>26.9) [<100]	>261 (>44.5) [<100]	>285 (>47.3) [<100]	>234 (>39.7) [<100]	
Unknown	<11 (<0.5)	<11 (<1.9) [<100]	<11 (<1.9) [<100]	<11 (<1.8) [<100]	<11 (<1.9) [<100]	
Kurman epithelial type						
I	400 (17.0)	80 (13.8) [20.0]	126 (21.5) [31.5]	109 (18.1) [27.3]	85 (14.4) [21.3]	.001
II	1959 (83.0)	501 (86.2) [25.6]	460 (78.5) [23.5]	493 (81.9) [25.2]	505 (85.6) [25.8]	
Earliest symptomatic presentation to ED						
Yes	485 (20.6)	200 (34.4) [41.2]	91 (15.5) [18.8]	95 (15.8) [19.6]	99 (16.8) [20.4]	<.001
No	1874 (79.4)	381 (65.6) [20.3]	495 (84.5) [26.4]	507 (84.2) [27.1]	491 (83.2) [26.2]	
Only 1 visit with symptoms during interval^b^						
Yes	285 (12.1)	198 (34.1) [69.5]	37 (6.3) [13.0]	31 (5.1) [10.9]	19 (3.2) [6.7]	<.001
No	2074 (87.9)	383 (65.9) [18.5]	549 (93.7) [26.5]	571 (94.9) [27.5]	571 (96.8) [27.5]	
Initial presenting symptom category^c^						
Pelvic pain	232 (9.8)	65 (11.2) [28.0]	72 (12.3) [31.0]	63 (10.5) [27.2]	32 (5.4) [13.8]	<.001
Abdominal pain or tenderness	1355 (57.4)	373 (64.2) [27.5]	340 (58.0) [25.1]	332 (55.1) [24.5]	310 (52.5) [22.9]	<.001
Gastrointestinal symptoms	988 (41.9)	247 (42.5) [25.0]	181 (30.9) [18.3]	248 (41.2) [25.1]	312 (52.9) [31.6]	<.001
Pelvic or abdominal swelling	1015 (43.0)	474 (81.6) [46.7]	353 (60.2) [34.8]	156 (25.9) [15.4]	32 (5.4) [3.2]	<.001

Abbreviations: CCI, Charlson Comorbidity Index; ED, emergency department; SEER, Surveillance, Epidemiology, and End Results; SES, socioeconomic status.

^a^ Includes American Indian or Alaska Native, Asian American, Native Hawaiian or Other Pacific Islander, multiracial, or any race or ethnicity not otherwise specified.

^b^ Symptoms include those in categories of pelvic pain, abdominal pain and tenderness, gastrointestinal symptoms, and pelvic or abdominal swelling.

^c^ Patients may have had more than 1 initial presenting symptom category.

Abdominal pain or tenderness was the most common earliest presenting symptom (1355 [57.4%]), followed by pelvic or abdominal swelling (1015 [43.0%]), gastrointestinal symptoms (988 [41.9%]), and pelvic pain (232 [9.8%]). In the sensitivity analysis including SSDs among 2450 patients, health care interactions for gastrointestinal disorder diagnoses defined the start of the interval for 774 (31.6%) and menopausal symptoms for 137 (5.6%) (eTable 4 in Supplement 1). The prevalence of pelvic or abdominal swelling as the initial symptom category declined markedly across interval quartiles, from 474 of 581 (81.6%) in the shortest quartile to 32 of 590 (5.4%) in the longest.

**Figure 2.**
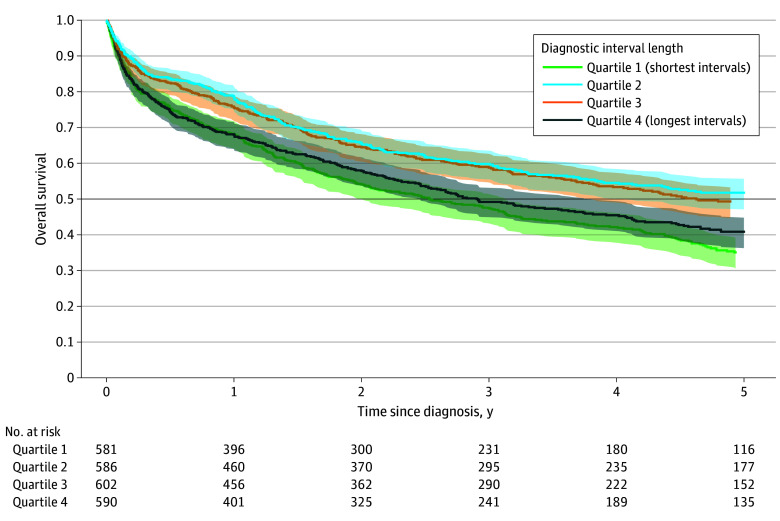
Kaplan-Meier Unadjusted Survival Curves by Quartile of the Diagnostic Interval Length

**Figure 3.**
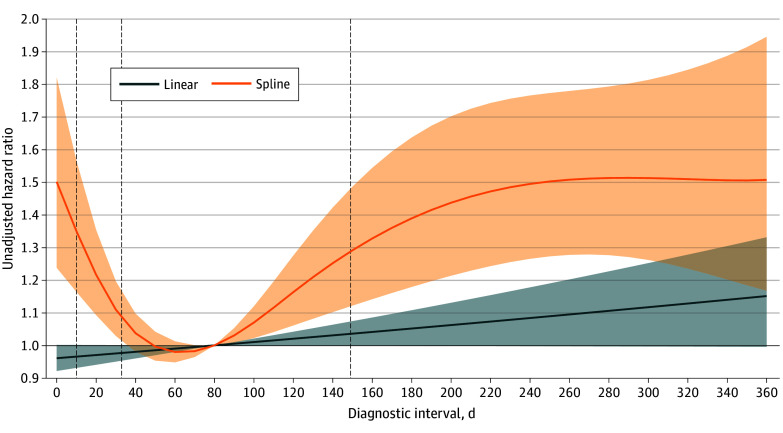
Linear and Restricted Cubic Spline Plot Showing the Unadjusted Association Between Diagnostic Interval and Overall Survival Hazard ratios were calculated using a diagnostic interval length of 80 days as the reference group; shaded areas indicate 95% CIs. Quartiles of the diagnostic interval are marked by vertical dashed lines.

**Figure 4.**
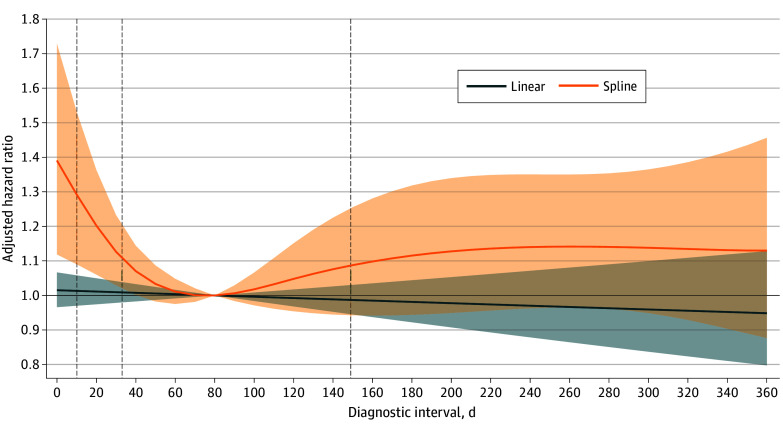
Linear and Restricted Cubic Spline Plot Showing the Adjusted Association Between Diagnostic Interval and Overall Survival Hazard ratios were calculated using a diagnostic interval length of 80 days as the reference group; shaded areas indicate 95% CIs. Quartiles of the diagnostic interval are marked by vertical dashed lines. Hazard ratios were adjusted for age at diagnosis, Charlson Comorbidity Index categories, quintiles of neighborhood-level socioeconomic status, epithelial type, categorical year of diagnosis, insurance payer, whether the initial presentation was to the emergency department, and symptom categories at the earliest presentation.

Unadjusted survival was highest in the second diagnostic interval quartile, with the median survival not reached within 5 years of follow-up (lower 95% CI bound, 4.1 years) (Figure 2). The median survival in the third quartile was 4.6 years (95% CI, 3.8 to >5 years), compared with 2.9 years (95% CI, 2.5-3.8 years) in the longest quartile and 2.5 years (95% CI, 2.0-3.1 years) in the shortest quartile. Linear modeling of the diagnostic interval term yielded unadjusted 5-year mortality HR estimates near the null value (Figure 3). In contrast, restricted cubic spline modeling revealed a U-shaped association between diagnostic interval length and mortality (Figure 3). Adjusted for potential confounders, the HR for a 100-day increase in diagnostic interval length using the linear model was 0.98 (95% CI, 0.92-1.04). With spline terms, a U-shape was still observed in the adjusted model, with an interval of 80 days having the lowest hazard of mortality compared with shorter or longer intervals (Figure 4). Specifically, relative to an interval of 80 days, the HRs for an interval of 10 days and 360 days were 1.29 (95% CI, 1.09-1.53) and 1.13 (95% CI, 0.88-1.46), respectively. Adjustment for stage (a potential intermediate) did not materially alter this association (eFigure 2 in Supplement 1), and results were similar in the sensitivity analysis including SSDs and urinary symptoms, with the lowest hazard at approximately 60 days (eFigure 1 in Supplement 1).

## Discussion

In the absence of a biologically plausible mechanism by which more rapid diagnosis would increase mortality, the observed U-shaped association suggests residual confounding by symptom severity, whereby patients with the most aggressive OCs are diagnosed more quickly but have poorer prognosis. Flexible nonlinear modeling revealed that patients with the longest diagnostic intervals also had increased mortality compared with those with intermediate intervals, consistent with a biologically plausible process in which diagnostic delays allowed disease progression before treatment. This association was obscured in models with a linear term, suggesting that prior studies using linear approaches could have underestimated harms associated with diagnostic delay.^8^

Patients with the shortest intervals in our study were likely diagnosed rapidly due to severe symptoms from aggressive underlying disease. Consistent with this idea, these patients were more likely to have distant-stage disease, aggressive Kurman epithelial types (type II), ED presentation, and pelvic or abdominal swelling at the initial presentation, a clearer indication of a potential malignant neoplasm.^27^ Patients in the longest quartile had a high prevalence of distant-stage disease but were less likely to present with swelling, suggesting fewer overt symptoms initially followed by disease progression over time before diagnosis. This is further supported by the fact that 198 patients in the shortest quartile (34.1%) were diagnosed after only 1 visit for the included symptoms compared with only 19 (3.2%) in the longest quartile, indicating that patients in the longest quartile may have had to seek more care before OC was suspected.

Patients in the second diagnostic interval quartile had the most favorable outcomes, with median survival at least 2 years longer than those in the shortest and longest quartiles. These patients were diagnosed relatively early but with fewer indicators of advanced disease. They were also less likely to reside in socioeconomically deprived neighborhoods or to belong to racial and ethnic minority groups, factors associated with fewer barriers to timely quality care. They were also younger, which may have prompted greater clinical concern at symptom presentation. Interventions that similarly support earlier diagnosis in the absence of advanced disease, such as predictive models for OC among patients with early abdominal and gastrointestinal symptoms, may therefore improve outcomes.

Although some have suggested that investment in symptom-based early detection may be futile,^28^ our findings indicate that symptomatic patients diagnosed up to 12 months after their initial presentation experienced poorer survival than those diagnosed within 30 to 200 days. These longer intervals were most often defined by abdominal or gastrointestinal symptoms, which are commonly attributed to benign conditions and may delay suspicion of OC. Indeed, more than 30% of patients in our sensitivity analysis had gastrointestinal disorder diagnoses defining the start of the interval, suggesting that diagnostic workup for common conditions may contribute to delayed cancer detection.

Several large trials of OC screening in asymptomatic populations^29,30^ have shown no benefit to screening for OC among those who do not yet have symptoms. However, many of these were initiated more than 2 decades ago when OC treatment paradigms differed from those in current practice.^29,31^ Additionally, these trials do not address whether diagnostic delay following symptom presentation affects outcomes, particularly given differences in ultrasonographic sensitivity for asymptomatic vs symptomatic disease.^32^ Moreover, mortality effects of untreated cancer progression during advanced symptomatic stages may differ from those during early, asymptomatic stages.

As highlighted in a systematic review,^8^ 19 studies examined the association between diagnostic timing and stage or survival of OC, with most reporting no association or even worse survival with faster diagnosis. These studies lacked nonlinear methods that would allow the wait time paradox to be visualized.^8^ However, our results are similar to a prior small study in Canada also using restricted cubic splines,^33^ which suggested that among patients with late-stage OC, those with intervals of approximately 80 days had better survival than patients with shorter or longer intervals, indicating that survival was poorer in patients diagnosed both early and late. Similarly, a multicountry study^34^ identified a U-shaped association between stage and the secondary care interval, or time from patient referral to diagnosis. Our study with nearly 4 times as many patients provides further evidence of the role of the wait time paradox in analyses of OC diagnosis.

### Limitations

This study has several limitations. Despite adjustment for measured indicators of disease severity, the shortest intervals continued to appear associated with higher mortality, suggesting persistent residual confounding. Methods addressing unmeasured confounding, such as instrumental variable analysis, may be needed to fully disentangle the wait time paradox.^35^ Nonetheless, our approach allows this confounding to be visualized, while distinguishing effects of very long diagnostic intervals from those of shorter intervals.

Although our sample is larger than those of prior nonlinear studies,^33,34^ repetition in larger samples may improve precision. As a state-based study, our findings may not generalize to all settings; however, our data source captures more than 80% of patients with any cancer in North Carolina and includes a greater proportion of patients from racial and ethnic minority groups and premenopausal patients than the national OC screening trial.^29,36^ Thus, our population is largely representative of insured women diagnosed with OC in North Carolina, while our requirement of continuous insurance enrollment supports the internal validity of our results by minimizing misclassification related to care not included in insurance claims. To maximize our sample, we only required 1 year of continuous insurance enrollment in our cohort and did not examine claims earlier than a year before diagnosis. However, some patients had diagnostic intervals as long as 365 days, indicating the actual length of the longest diagnostic intervals may be truncated by the 1-year lookback window, although observed maximum intervals exceeded those reported in most prior studies. An in-depth analysis of the diagnostic window should be conducted to determine the earliest time that use of health care services for a symptom could be related to OC.^37,38,39^ We also did not fully characterize the clinical setting of the initial presentation other than the ED or investigate the role of distance to care, although this has been evaluated in prior studies.^5,40,41,42^ Some symptom-related health care encounters may not have been included without relevant symptom-related *ICD* codes. To mitigate this, we used a more inclusive definition for the start of the diagnostic interval in a sensitivity analyses, with evidence of the wait time paradox still observed. Contrarily, we were unable to verify whether the included *ICD* codes were truly associated with the underlying OC rather than another illness or chronic condition. In our sensitivity analysis including urinary symptoms and SSDs, we found that HRs for patients with intervals longer than 180 days were somewhat lower than those for intervals of 160 days. This suggests including broader, more nonspecific claims to define the start of the interval could cause mismeasurement and exaggeration of the interval if claims were unrelated to OC, making the longest intervals appear less harmful. Additionally, some studies of the diagnostic interval adjust for stage, which may be a mediator of the interval-survival association; earlier diagnosis may detect OC at a more localized stage, resulting in better survival. We did not adjust for stage in our main analysis, although adjustment in a sensitivity analysis demonstrated minimal impact.

## Conclusions

In this cohort study of patients with OC in North Carolina, nonlinear modeling revealed patterns consistent with the wait time paradox while suggesting that earlier diagnosis may potentially improve outcomes for some patients. Prior studies reporting no association between diagnostic timeliness and survival may reflect methodologic limitations rather than biological reality. Larger studies using nuanced analytical approaches, along with investment in clinical tools to improve diagnostic timing, may improve patient outcomes for this aggressive cancer.

## Notes

Supplement 1. **eTable 1.** Codes Used to Identify Surgical Procedures Associated With Ovarian Cancer (OC) Biopsy to Define the Index Date Marking the Earliest Clinical Suspicion of OC **eTable 2.** *International Classification of Diseases* Codes Used to Identify Health Care Encounters Related to Potential Ovarian Cancer Symptom Categories **eTable 3.** *International Classification of Diseases* Codes Used to Identify Health Care Encounters Related to Symptomatically Similar Diseases (SSDs) **eTable 4.** Diagnostic Interval Length Using Symptomatically Similar Diseases (SSDs) and Symptoms, Including Urinary, to Define the Start of the Interval, Stratified by Initial Presenting Symptoms or SSDs **eFigure 1.** Sensitivity Analysis Results Including Symptomatically Similar Diseases (SSDs) to Define the Start of the Diagnostic Interval **eFigure 2.** Sensitivity Analysis Adjusting for Stage in the Association Between Diagnostic Interval Length and Survival

Supplement 2.
Data Sharing Statement
