# Supplementary material for: Diagnostic Timing and Ovarian Cancer Survival
Source: JAMA Netw Open. 2026 Mar 27;9(3):e262434. doi: 10.1001/jamanetworkopen.2026.2434 (PMC13032158; doi:10.1001/jamanetworkopen.2026.2434)
Supplement: Supplement 1. — eTable 1. Codes Used to Identify Surgical Procedures Associated With Ovarian Cancer (OC) Biopsy to Define the Index Date Marking the Earliest Clinical Suspicion of OC eTable 2. International Classification of Diseases Codes Used to Identify Health Care Encounters Related to Potential Ovarian Cancer Symptom Categories eTable 3. International Classification of Diseases Codes Used to Identify Health Care Encounters Related to Symptomatically Similar Diseases (SSDs) eTable 4. Diagnostic Interval Length Using Symptomatically Similar Diseases (SSDs) and Symptoms, Including Urinary, to Define the Start of the Interval, Stratified by Initial Presenting Symptoms or SSDs eFigure 1. Sensitivity Analysis Results Including Symptomatically Similar Diseases (SSDs) to Define the Start of the Diagnostic Interval eFigure 2. Sensitivity Analysis Adjusting for Stage in the Association Between Diagnostic Interval Length and Survival [file jamanetwopen-e262434-s001.pdf]

Supplementary Online Content

Soppe SE, Kuo TM, Bae-Jump VL, et al. Diagnostic timing and ovarian cancer survival. *JAMA Netw Open.* 2026;9(3):e262434. doi:10.1001/jamanetworkopen.2026.2434

eTable 1. Codes Used to Identify Surgical Procedures Associated With Ovarian Cancer (OC) Biopsy to Define the Index Date Marking the Earliest Clinical Suspicion of OC

eTable 2. *International Classification of Diseases* Codes Used to Identify Health Care Encounters Related to Potential Ovarian Cancer Symptom Categories

eTable 3. *International Classification of Diseases* Codes Used to Identify Health Care Encounters Related to Symptomatically Similar Diseases (SSDs)

eTable 4. Diagnostic Interval Length Using Symptomatically Similar Diseases (SSDs) and Symptoms, Including Urinary, to Define the Start of the Interval, Stratified by Initial Presenting Symptoms or SSDs

eFigure 1. Sensitivity Analysis Results Including Symptomatically Similar Diseases (SSDs) to Define the Start of the Diagnostic Interval

eFigure 2. Sensitivity Analysis Adjusting for Stage in the Association Between Diagnostic Interval Length and Survival

This supplementary material has been provided by the authors to give readers additional information about their work.

eTable 1. Codes Used to Identify Surgical Procedures Associated With Ovarian Cancer (OC) Biopsy to Define the Index Date Marking the Earliest Clinical Suspicion of OC

Procedure type	Code type	Code
Excision	ICD-9	652
	ICD-9	6521
	ICD-9	6522
	ICD-9	6523
	ICD-9	6524
	ICD-9	6525
	ICD-9	6529
	ICD-10	0U900ZZ
	ICD-10	0U903ZZ
	ICD-10	0U910ZZ
	ICD-10	0U913ZZ
	ICD-10	0U920ZZ
	ICD-10	0U923ZZ
	ICD-10	0UB07ZZ
	ICD-10	0UB08ZZ
	ICD-10	0UB17ZZ
	ICD-10	0UB18ZZ
	ICD-10	0UB23ZZ
	ICD-10	0UB27ZZ
	ICD-10	0UB28ZZ
	ICD-10	0U904ZZ
	ICD-10	0U914ZZ
	ICD-10	0U924ZZ
	ICD-10	0U504ZZ
	ICD-10	0U514ZZ
	ICD-10	0U524ZZ
	ICD-10	0UB04ZZ
	ICD-10	0UB14ZZ
	ICD-10	0UB24ZZ
	ICD-10	0U500ZZ
	ICD-10	0U503ZZ
	ICD-10	0U508ZZ
	ICD-10	0U510ZZ
	ICD-10	0U513ZZ
	ICD-10	0U518ZZ
	ICD-10	0U520ZZ

	ICD-10	0U523ZZ
	ICD-10	0U528ZZ
	ICD-10	0U800ZZ
	ICD-10	0U803ZZ
	ICD-10	0U810ZZ
	ICD-10	0U813ZZ
	ICD-10	0U820ZZ
	ICD-10	0U823ZZ
	ICD-10	0UB00ZZ
	ICD-10	0UB03ZZ
	ICD-10	0UB10ZZ
	ICD-10	0UB13ZZ
	ICD-10	0UB20ZZ
	ICD-9	7032
	ICD-10	0U5F0ZZ
	ICD-10	0U5F3ZZ
	ICD-10	0U5F4ZZ
	ICD-10	0U5F7ZZ
	ICD-10	0U5F8ZZ
	ICD-10	0UBF0ZZ
	ICD-10	0UBF3ZZ
	ICD-10	0UBF4ZZ
	ICD-10	0UBF7ZZ
	ICD-10	0UBF8ZZ
	CPT	56303
Hysterectomy	ICD-9	683
	ICD-9	6831
	ICD-9	6839
	ICD-9	684
	ICD-9	6841
	ICD-9	6849
	ICD-9	685
	ICD-9	6851
	ICD-9	6859
	ICD-9	686
	ICD-9	6861
	ICD-9	6869
	ICD-9	687
	ICD-9	6871
	ICD-9	6879
	ICD-9	689

	ICD-10	0UT94ZL
	ICD-10	0UT90ZL
	ICD-10	0UT94ZZ
	ICD-10	0UT9FZL
	ICD-10	0UT97ZL
	ICD-10	0UT98ZL
	ICD-10	0UT40ZZ
	ICD-10	0UT90ZZ
	ICD-10	0UTC0ZZ
	ICD-10	0UT44ZZ
	ICD-10	0UT9FZZ
	ICD-10	0UTC4ZZ
	ICD-10	0UT47ZZ
	ICD-10	0UT48ZZ
	ICD-10	0UT97ZZ
	ICD-10	0UT98ZZ
	ICD-10	0UTC7ZZ
	ICD-10	0UTC8ZZ
	CPT	56308
	CPT	58150
	CPT	58152
	CPT	58180
	CPT	58200
	CPT	58210
	CPT	58262
	CPT	58263
	CPT	58951
	CPT	58953
	CPT	58954
Laparoscopy	ICD-9	5421
	CPT	38120
	CPT	38570
	CPT	38571
	CPT	38572
	CPT	43280
	CPT	43651
	CPT	43652
	CPT	43653
	CPT	44200
	CPT	44201
	CPT	44209

	CPT	49321
	CPT	49322
Laparotomy	CPT	58960
	ICD-9	541
	ICD-9	5411
	ICD-9	5419
	CPT	49000
Needle biopsy	ICD-9	5424
	CPT	49180
Omentectomy	ICD-9	544
	ICD-10	0D5U0ZZ
	ICD-10	0D5U3ZZ
	ICD-10	0D5U4ZZ
	ICD-10	0D5V0ZZ
	ICD-10	0D5V3ZZ
	ICD-10	0D5V4ZZ
	ICD-10	0D5W0ZZ
	ICD-10	0D5W3ZZ
	ICD-10	0D5W4ZZ
	ICD-10	0DBU0ZZ
	ICD-10	0DBU3ZZ
	ICD-10	0DBU4ZZ
	ICD-10	0DBV0ZZ
	ICD-10	0DBV3ZZ
	ICD-10	0DBV4ZZ
	ICD-10	0DBW0ZZ
	ICD-10	0DBW3ZZ
	ICD-10	0DBW4ZZ
	ICD-10	0DTU0ZZ
	ICD-10	0DTU4ZZ
	ICD-10	0WBH0ZZ
	ICD-10	0WBH3ZZ
	ICD-10	0WBH4ZZ
Oophorectomy	ICD-9	653
	ICD-9	6531
	ICD-9	6539
	ICD-10	0UT07ZZ
	ICD-10	0UT08ZZ
	ICD-10	0UT0FZZ
	ICD-10	0UT17ZZ
	ICD-10	0UT18ZZ

	ICD-10	0UT1FZZ
	ICD-9	654
	ICD-9	6541
	ICD-9	6549
	ICD-9	655
	ICD-9	6551
	ICD-9	6552
	ICD-9	6553
	ICD-9	6554
	ICD-10	0UT27ZZ
	ICD-10	0UT28ZZ
	ICD-10	0UT2FZZ
	CPT	58940
	CPT	58943
Paracentesis	CPT	49082
	CPT	49083
Pelvic exenteration	ICD-9	688
	ICD-10	0UB70ZZ
	ICD-10	0UB73ZZ
	ICD-10	0UB74ZZ
	ICD-10	0UB77ZZ
	ICD-10	0UB78ZZ
	ICD-10	0UB50ZZ
	ICD-10	0UB53ZZ
	ICD-10	0UB54ZZ
	ICD-10	0UB57ZZ
	ICD-10	0UB58ZZ
	ICD-10	0UB60ZZ
	ICD-10	0UB63ZZ
	ICD-10	0UB64ZZ
	ICD-10	0UB67ZZ
	ICD-10	0UB68ZZ
	CPT	58240
Resection	CPT	58920
Salpingectomy	ICD-9	6663
	ICD-9	6669
Salpingo-oophorectomy	ICD-9	656
	ICD-9	6561
	ICD-9	6562
	ICD-9	6563
	ICD-9	6564

	ICD-10	0UT20ZZ
	ICD-10	0UT70ZZ
	ICD-10	0UT00ZZ
	ICD-10	0UT10ZZ
	ICD-10	0UT50ZZ
	ICD-10	0UT60ZZ
	ICD-10	0UT24ZZ
	ICD-10	0UT74ZZ
	ICD-10	0UT04ZZ
	ICD-10	0UT14ZZ
	ICD-10	0UT54ZZ
	ICD-10	0UT64ZZ
	CPT	56307
	CPT	57531
	CPT	58720
	CPT	58950
	CPT	58952

eTable 2. ***International Classification of Diseases*** **Codes Used to Identify Health Care Encounters Related to Potential Ovarian Cancer Symptom Categories**

Symptom Category	Code Type	Code	Symptom Detail
Abdominal or pelvic swelling	ICD10	R1906	Epigastric swelling, mass or lump
	ICD10	R1907	Generalized intra-abdominal and pelvic swelling, mass and lump
	ICD10	R190	Intra-abdominal and pelvic swelling, mass and lump
	ICD10	R1900	Intra-abdominal and pelvic swelling, mass and lump, unspecified site
	ICD10	R1904	Left lower quadrant abdominal swelling, mass and lump
	ICD10	R1902	Left upper quadrant abdominal swelling, mass and lump
	ICD10	R188	Other ascites
	ICD10	R1909	Other intra-abdominal and pelvic swelling, mass and lump
	ICD10	R1905	Periumbilic swelling, mass or lump
	ICD10	R1903	Right lower quadrant abdominal swelling, mass and lump
	ICD10	R1901	Right upper quadrant abdominal swelling, mass and lump
	ICD9	7893	Abdominal or pelvic swelling, mass, or lump
	ICD9	78936	Abdominal or pelvic swelling, mass, or lump; epigastric
	ICD9	78937	Abdominal or pelvic swelling, mass, or lump; generalized
	ICD9	78934	Abdominal or pelvic swelling, mass, or lump; left lower quadrant
	ICD9	78932	Abdominal or pelvic swelling, mass, or lump; left upper quadrant
	ICD9	78939	Abdominal or pelvic swelling, mass, or lump; other specified site
	ICD9	78935	Abdominal or pelvic swelling, mass, or lump; periumbilic
	ICD9	78933	Abdominal or pelvic swelling, mass, or lump; right lower quadrant
	ICD9	78931	Abdominal or pelvic swelling, mass, or lump; right upper quadrant
	ICD9	78930	Abdominal or pelvic swelling, mass, or lump; unspecified site
	ICD9	78959	Other ascites
	ICD9	56882	Peritoneal effusion (chronic)
Abdominal pain/tenderness	ICD10	R100	Acute abdomen
	ICD10	R1013	Epigastric pain
	ICD10	R1032	Left lower quadrant pain
	ICD10	R1012	Left upper quadrant pain
	ICD10	R1030	Lower abdominal pain, unspecified

	ICD10	R1033	Periumbilic pain
	ICD10	R1031	Right lower quadrant pain
	ICD10	R1011	Right upper quadrant pain
	ICD10	R109	Unspecified abdominal pain
	ICD10	R1010	Upper abdominal pain, unspecified
	ICD9	78906	Abdominal pain, epigastric
	ICD9	78907	Abdominal pain, generalized
	ICD9	78904	Abdominal pain, left lower quadrant
	ICD9	78902	Abdominal pain, left upper quadrant
	ICD9	78909	Abdominal pain, other specified site
	ICD9	78905	Abdominal pain, periumbilic
	ICD9	78903	Abdominal pain, right lower quadrant
	ICD9	78901	Abdominal pain, right upper quadrant
	ICD9	78900	Abdominal pain, unspecified site
	ICD10	R10819	Abdominal tenderness, unspecified site
	ICD10	R10816	Epigastric abdominal tenderness
	ICD10	R10826	Epigastric rebound abdominal tenderness
	ICD10	R10817	Generalized abdominal tenderness
	ICD10	R10827	Generalized rebound abdominal tenderness
	ICD10	R10814	Left lower quadrant abdominal tenderness
	ICD10	R10824	Left lower quadrant rebound abdominal tenderness
	ICD10	R10812	Left upper quadrant abdominal tenderness
	ICD10	R10822	Left upper quadrant rebound abdominal tenderness
	ICD10	R10815	Periumbilic abdominal tenderness
	ICD10	R10825	Periumbilic rebound abdominal tenderness
	ICD10	R10829	Rebound abdominal tenderness, unspecified site
	ICD10	R10813	Right lower quadrant abdominal tenderness
	ICD10	R10823	Right lower quadrant rebound abdominal tenderness
	ICD10	R10811	Right upper quadrant abdominal tenderness
	ICD10	R10821	Right upper quadrant reboud abdominal tenderness
	ICD9	78966	Abdominal tenderness, epigastric
	ICD9	78967	Abdominal tenderness, generalized
	ICD9	78964	Abdominal tenderness, left lower quadrant
	ICD9	78962	Abdominal tenderness, left upper quadrant
	ICD9	78969	Abdominal tenderness, other specified site
	ICD9	78965	Abdominal tenderness, periumbilic
	ICD9	78963	Abdominal tenderness, right lower quadrant
	ICD9	78961	Abdominal tenderness, right upper quadrant
	ICD9	78960	Abdominal tenderness, unspecified site

Gastrointestinal symptoms	ICD10	R140	Abdominal distension (gaseous)
	ICD10	K3183	Achlorhydria
	ICD10	R630	Anorexia
	ICD10	R194	Change in bowel habit
	ICD10	K5900	Constipation, unspecified
	ICD10	R197	Diarrhea unspecified
	ICD10	K319	Disease of stomach and duodenum, unspecified
	ICD10	R142	Eructation
	ICD10	R143	Flatulence
	ICD10	R141	Gas pain
	ICD10	R110	Nausea
	ICD10	K5909	Other constipation
	ICD10	K3189	Other diseases of stomach and duodenum
	ICD10	R198	Other specified symptoms and signs involving the digestive system and abdomen
	ICD10	K5902	Outlet dysfunction constipation
	ICD10	K5901	Slow transit constipation
	ICD9	5360	Achlorhydria
	ICD9	7830	Anorexia
	ICD9	56400	Constipation, unspecified
	ICD9	78791	Diarrhea
	ICD9	7873	Flatulence, eructation, and gas pain
	ICD9	78702	Nausea alone
	ICD9	56409	Other constipation
	ICD9	7879	Other symptoms involving digestive system
	ICD9	78799	Other symptoms involving digestive system
	ICD9	56402	Outlet dysfunction constipation
	ICD9	56401	Slow transit constipation
	ICD9	5369	Unspecified functional disorder of stomach
Pelvic pain	ICD10	N9412	Deep dyspareunia
	ICD10	N940	Dyspareunia
	ICD10	N9489	Other specified conditions associated with female genital organs and menstrual cycle
	ICD10	N9419	Other specified dyspareunia
	ICD10	R102	Pelvic and perineal pain
	ICD10	N9411	Superficial (introital) dyspareunia
	ICD10	N949	Unspecified condition associated with female genital organs and menstrual cycle
	ICD10	N9410	Unspecified dyspareunia
	ICD9	6250	Dyspareunia
	ICD9	6255	Pelvic congestion syndrome
	ICD9	6259	Unspecified symptom associated with female genital organs

Urinary symptoms	ICD10	N39491	Coital incontinence
	ICD10	N3945	Continuous leakage
	ICD10	R300	Dysuria
	ICD10	R350	Frequency of micturition
	ICD10	N3942	Incontinence without sensory awareness
	ICD10	N3946	Mixed incontinence
	ICD10	R351	Nocturia
	ICD10	N3944	Nocturnal enuresis
	ICD10	R358	Other polyuria
	ICD10	N3949	Other specified urinary incontinence
	ICD10	N39498	Other specified urinary incontinence
	ICD10	N39490	Overflow incontinence
	ICD10	R309	Painful micturition, unspecified
	ICD10	N39492	Postural (urinary) incontinence
	ICD10	N3943	Post-void dribbling
	ICD10	R32	Unspecified urinary incontinence
	ICD10	N3941	Urge incontinence
	ICD9	78837	Continuous leakage
	ICD9	7881	Dysuria
	ICD9	7884	Frequency of urination and polyuria
	ICD9	78834	Incontinence without sensory awareness
	ICD9	78833	Mixed incontinence (female) (male)
	ICD9	78843	Nocturia
	ICD9	78836	Nocturnal enuresis
	ICD9	78839	Other urinary incontinence
	ICD9	78842	Polyuria
	ICD9	78835	Post-void dribbling
	ICD9	78831	Urge incontinence
	ICD9	78841	Urinary frequency
	ICD9	7883	Urinary incontinence
	ICD9	78830	Urinary incontinence, unspecified
	IDC9	78838	Overflow incontinence

eTable 3. *International Classification of Diseases* Codes Used to Identify Health Care Encounters Related to Symptomatically Similar Diseases (SSDs)

Category	Code Type	Code	Symptom Detail
Crohn's disease	ICD10	K50	Crohn's disease [regional enteritis]
	ICD10	K500	Crohn's disease of small intestine
	ICD10	K50014	Crohn's disease of small intestine with abscess
	ICD10	K5001	Crohn's disease of small intestine with complications
	ICD10	K50013	Crohn's disease of small intestine with fistula
	ICD10	K50012	Crohn's disease of small intestine with intestinal obstruction
	ICD10	K50018	Crohn's disease of small intestine with other complication
	ICD10	K50011	Crohn's disease of small intestine with rectal bleeding
	ICD10	K50019	Crohn's disease of small intestine with unspecified complications
	ICD10	K5000	Crohn's disease of small intestine without complications
	ICD9	5551	Large intestine
	ICD9	555	Regional enteritis
	ICD9	5550	Small intestine
	ICD9	5552	Small intestine with large intestine
	ICD9	5559	Unspecified site
Diverticular disease	ICD10	K575	Diverticular disease of both small and large intestine without perforation or abscess
	ICD10	K57	Diverticular disease of intestine
	ICD10	K579	Diverticular disease of intestine, part unspecified, without perforation or abscess
	ICD10	K573	Diverticular disease of large intestine without perforation or abscess
	ICD10	K571	Diverticular disease of small intestine without perforation or abscess
	ICD10	K574	Diverticulitis of both small and large intestine with perforation and abscess
	ICD10	K5741	Diverticulitis of both small and large intestine with perforation and abscess with bleeding
	ICD10	K5740	Diverticulitis of both small and large intestine with perforation and abscess without bleeding
	ICD10	K5753	Diverticulitis of both small and large intestine without perforation or abscess with bleeding
	ICD10	K5752	Diverticulitis of both small and large intestine without perforation or abscess without bleeding
	ICD10	K578	Diverticulitis of intestine, part unspecified, with perforation and abscess
	ICD10	K5781	Diverticulitis of intestine, part unspecified, with perforation and abscess with bleeding
	ICD10	K5780	Diverticulitis of intestine, part unspecified, with perforation and abscess without bleeding
	ICD10	K5793	Diverticulitis of intestine, part unspecified, without perforation or abscess with bleeding

	ICD10	K5792	Diverticulitis of intestine, part unspecified, without perforation or abscess without bleeding
	ICD10	K572	Diverticulitis of large intestine with perforation and abscess
	ICD10	K5721	Diverticulitis of large intestine with perforation and abscess with bleeding
	ICD10	K5720	Diverticulitis of large intestine with perforation and abscess without bleeding
	ICD10	K5733	Diverticulitis of large intestine without perforation or abscess with bleeding
	ICD10	K5732	Diverticulitis of large intestine without perforation or abscess without bleeding
	ICD10	K570	Diverticulitis of small intestine with perforation and abscess
	ICD10	K5701	Diverticulitis of small intestine with perforation and abscess with bleeding
	ICD10	K5700	Diverticulitis of small intestine with perforation and abscess without bleeding
	ICD10	K5713	Diverticulitis of small intestine without perforation or abscess with bleeding
	ICD10	K5712	Diverticulitis of small intestine without perforation or abscess without bleeding
	ICD10	K5751	Diverticulosis of both small and large intestine without perforation or abscess with bleeding
	ICD10	K5750	Diverticulosis of both small and large intestine without perforation or abscess without bleeding
	ICD10	K5791	Diverticulosis of intestine, part unspecified, without perforation or abscess with bleeding
	ICD10	K5790	Diverticulosis of intestine, part unspecified, without perforation or abscess without bleeding
	ICD10	K5731	Diverticulosis of large intestine without perforation or abscess with bleeding
	ICD10	K5730	Diverticulosis of large intestine without perforation or abscess without bleeding
	ICD10	K5711	Diverticulosis of small intestine without perforation or abscess with bleeding
	ICD10	K5710	Diverticulosis of small intestine without perforation or abscess without bleeding
	ICD9	5621	Diverticula of colon
	ICD9	562	Diverticula of intestine
	ICD9	5620	Diverticula of small intestine
	ICD9	56213	Diverticulitis of colon with hemorrhage
	ICD9	56211	Diverticulitis of colon without mention of hemorrhage
	ICD9	56201	Diverticulitis of small intestine (without mention of hemorrhage)
	ICD9	56203	Diverticulitis of small intestine with hemorrhage
	ICD9	56210	Diverticulosis of colon (without mention of hemorrhage)
	ICD9	56212	Diverticulosis of colon with hemorrhage
	ICD9	56200	Diverticulosis of small intestine (without mention of hemorrhage)
	ICD9	56202	Diverticulosis of small intestine with hemorrhage

Gastritis	ICD10	K290	Acute gastritis
	ICD10	K2901	Acute gastritis with bleeding
	ICD10	K2900	Acute gastritis without bleeding
	ICD10	K292	Alcoholic gastritis
	ICD10	K2921	Alcoholic gastritis with bleeding
	ICD10	K2920	Alcoholic gastritis without bleeding
	ICD10	K294	Chronic atrophic gastritis
	ICD10	K2941	Chronic atrophic gastritis with bleeding
	ICD10	K2940	Chronic atrophic gastritis without bleeding
	ICD10	K293	Chronic superficial gastritis
	ICD10	K2931	Chronic superficial gastritis with bleeding
	ICD10	K2930	Chronic superficial gastritis without bleeding
	ICD10	K5281	Eosinophilic gastritis or gastroenteritis
	ICD10	K29	Gastritis and duodenitis
	ICD10	K297	Gastritis, unspecified
	ICD10	K2971	Gastritis, unspecified, with bleeding
	ICD10	K2970	Gastritis, unspecified, without bleeding
	ICD10	K52	Other and unspecified noninfective gastroenteritis and colitis
	ICD10	K296	Other gastritis
	ICD10	K2961	Other gastritis with bleeding
	ICD10	K2960	Other gastritis without bleeding
	ICD10	K528	Other specified noninfective gastroenteritis and colitis
	ICD10	K295	Unspecified chronic gastritis
	ICD10	K2951	Unspecified chronic gastritis with bleeding
	ICD10	K2950	Unspecified chronic gastritis without bleeding
	ICD9	5350	Acute gastritis
	ICD9	53501	Acute gastritis with hemorrhage
	ICD9	53500	Acute gastritis without mention of hemorrhage
	ICD9	5353	Alcoholic gastritis
	ICD9	53531	Alcoholic gastritis with hemorrhage
	ICD9	53530	Alcoholic gastritis without mention of hemorrhage
	ICD9	5351	Atrophic gastritis
	ICD9	53511	Atrophic gastritis with hemorrhage
	ICD9	53510	Atrophic gastritis without mention of hemorrhage
	ICD9	5357	Eosinophilic gastritis
	ICD9	53571	Eosinophilic gastritis with hemorrhage

	ICD9	53570	Eosinophilic gastritis without mention of hemorrhage
	ICD9	5352	Gastric mucosal hypertrophy
	ICD9	535	Gastritis and duodenitis
	ICD9	5354	Other specified gastritis
	ICD9	53541	Other specified gastritis with hemorrhage
	ICD9	53540	Other specified gastritis without mention of hemorrhage
	ICD9	5355	Unspecified gastritis and gastroduodenitis
	ICD9	53551	Unspecified gastritis and gastroduodenitis with hemorrhage
	ICD9	53550	Unspecified gastritis and gastroduodenitis without mention of hemorrhage
Gastroesophageal reflux disease (GERD)	ICD10	K209	Esophagitis, unspecified
	ICD10	K210	Gastro-esophageal reflux disease with esophagitis
	ICD10	K219	Gastro-esophageal reflux disease without esophagitis
	ICD10	R12	Heartburn
	ICD9	53081	Esophageal reflux
	ICD9	5301	Esophagitis
	ICD9	7871	Heartburn
	ICD9	53011	Reflux esophagitis
Irritable bowel syndrome (IBS)	ICD10	K58	Irritable bowel syndrome
	ICD10	K581	Irritable bowel syndrome with constipation
	ICD10	K580	Irritable bowel syndrome with diarrhea
	ICD10	K589	Irritable bowel syndrome without diarrhea
	ICD10	K582	Mixed irritable bowel syndrome
	ICD10	K588	Other irritable bowel syndrome
	ICD9	5641	Irritable bowel syndrome
Menopausal symptoms	ICD10	N951	Menopausal and female climacteric states
	ICD10	N958	Other specified menopausal and perimenopausal disorders
	ICD10	N952	Postmenopausal atrophic vaginitis
	ICD10	N959	Unspecified menopausal and perimenopausal disorder
	ICD9	6278	Other specified menopausal and postmenopausal disorders
	ICD9	6273	Postmenopausal atrophic vaginitis
	ICD9	6272	Symptomatic menopausal or female climacteric states
	ICD9	6274	Symptomatic states associated with artificial menopause
	ICD9	6279	Unspecified menopausal and postmenopausal disorder
Ulcerative colitis	ICD10	K514	Inflammatory polyps of colon
	ICD10	K51414	Inflammatory polyps of colon with abscess
	ICD10	K5141	Inflammatory polyps of colon with complications
	ICD10	K51413	Inflammatory polyps of colon with fistula

	ICD10	K51412	Inflammatory polyps of colon with intestinal obstruction
	ICD10	K51418	Inflammatory polyps of colon with other complication
	ICD10	K51411	Inflammatory polyps of colon with rectal bleeding
	ICD10	K51419	Inflammatory polyps of colon with unspecified complications
	ICD10	K5140	Inflammatory polyps of colon without complications
	ICD10	K515	Left sided colitis
	ICD10	K51514	Left sided colitis with abscess
	ICD10	K5151	Left sided colitis with complications
	ICD10	K51513	Left sided colitis with fistula
	ICD10	K51512	Left sided colitis with intestinal obstruction
	ICD10	K51518	Left sided colitis with other complication
	ICD10	K51511	Left sided colitis with rectal bleeding
	ICD10	K51519	Left sided colitis with unspecified complications
	ICD10	K5150	Left sided colitis without complications
	ICD10	K518	Other ulcerative colitis
	ICD10	K51814	Other ulcerative colitis with abscess
	ICD10	K5181	Other ulcerative colitis with complications
	ICD10	K51813	Other ulcerative colitis with fistula
	ICD10	K51812	Other ulcerative colitis with intestinal obstruction
	ICD10	K51818	Other ulcerative colitis with other complication
	ICD10	K51811	Other ulcerative colitis with rectal bleeding
	ICD10	K51819	Other ulcerative colitis with unspecified complications
	ICD10	K5180	Other ulcerative colitis without complications
	ICD10	K510	Ulcerative (chronic) pancolitis
	ICD10	K51014	Ulcerative (chronic) pancolitis with abscess
	ICD10	K5101	Ulcerative (chronic) pancolitis with complications
	ICD10	K51013	Ulcerative (chronic) pancolitis with fistula
	ICD10	K51012	Ulcerative (chronic) pancolitis with intestinal obstruction
	ICD10	K51018	Ulcerative (chronic) pancolitis with other complication
	ICD10	K51011	Ulcerative (chronic) pancolitis with rectal bleeding
	ICD10	K51019	Ulcerative (chronic) pancolitis with unspecified complications
	ICD10	K5100	Ulcerative (chronic) pancolitis without complications
	ICD10	K512	Ulcerative (chronic) proctitis
	ICD10	K51214	Ulcerative (chronic) proctitis with abscess
	ICD10	K5121	Ulcerative (chronic) proctitis with complications
	ICD10	K51213	Ulcerative (chronic) proctitis with fistula
	ICD10	K51212	Ulcerative (chronic) proctitis with intestinal obstruction
	ICD10	K51218	Ulcerative (chronic) proctitis with other complication
	ICD10	K51211	Ulcerative (chronic) proctitis with rectal bleeding

	ICD10	K51219	Ulcerative (chronic) proctitis with unspecified complications
	ICD10	K5120	Ulcerative (chronic) proctitis without complications
	ICD10	K513	Ulcerative (chronic) rectosigmoiditis
	ICD10	K51314	Ulcerative (chronic) rectosigmoiditis with abscess
	ICD10	K5131	Ulcerative (chronic) rectosigmoiditis with complications
	ICD10	K51313	Ulcerative (chronic) rectosigmoiditis with fistula
	ICD10	K51312	Ulcerative (chronic) rectosigmoiditis with intestinal obstruction
	ICD10	K51318	Ulcerative (chronic) rectosigmoiditis with other complication
	ICD10	K51311	Ulcerative (chronic) rectosigmoiditis with rectal bleeding
	ICD10	K51319	Ulcerative (chronic) rectosigmoiditis with unspecified complications
	ICD10	K5130	Ulcerative (chronic) rectosigmoiditis without complications
	ICD10	K51	Ulcerative colitis
	ICD10	K519	Ulcerative colitis, unspecified
	ICD10	K51914	Ulcerative colitis, unspecified with abscess
	ICD10	K51913	Ulcerative colitis, unspecified with fistula
	ICD10	K51912	Ulcerative colitis, unspecified with intestinal obstruction
	ICD10	K51918	Ulcerative colitis, unspecified with other complication
	ICD10	K51911	Ulcerative colitis, unspecified with rectal bleeding
	ICD10	K51919	Ulcerative colitis, unspecified with unspecified complications
	ICD10	K5191	Ulcerative colitis, unspecified, with complications
	ICD10	K5190	Ulcerative colitis, unspecified, without complications
	ICD9	5565	Left-sided ulcerative (chronic) colitis
	ICD9	5568	Other ulcerative colitis
	ICD9	5564	Pseudopolyposis of colon
	ICD9	5560	Ulcerative (chronic) enterocolitis
	ICD9	5561	Ulcerative (chronic) ileocolitis
	ICD9	5562	Ulcerative (chronic) proctitis
	ICD9	5563	Ulcerative (chronic) proctosigmoiditis
	ICD9	556	Ulcerative colitis
	ICD9	5569	Ulcerative colitis, unspecified
	ICD9	5566	Universal ulcerative (chronic) colitis

eTable 4. Diagnostic Interval Length Using Symptomatically Similar Diseases (SSDs) and Symptoms, Including Urinary, to Define the Start of the Interval, Stratified by Initial Presenting Symptoms or SSDs

Initial presenting symptom(s) or SSDs to the healthcare system	N (%)	Diagnostic interval length (days)		
		Median	Mean	Std
Any	2,450 (100)	112.5	144.8	127.0
Pelvic or abdominal swelling	670 (27.3)	12	28.0	58.2
Pelvic pain	141 (5.8)	24	65.3	89.8
Abdominal pain or tenderness	904 (36.9)	32	92.9	112.1
Gastrointestinal symptoms	645 (26.3)	53	117.1	122.5
Urinary symptoms	406 (16.6)	205	189.2	122.8
Gastrointestinal disorder diagnosis	774 (31.6)	211	187.1	124.9
Menopausal symptom diagnosis	137 (5.6)	227	206.7	111.6

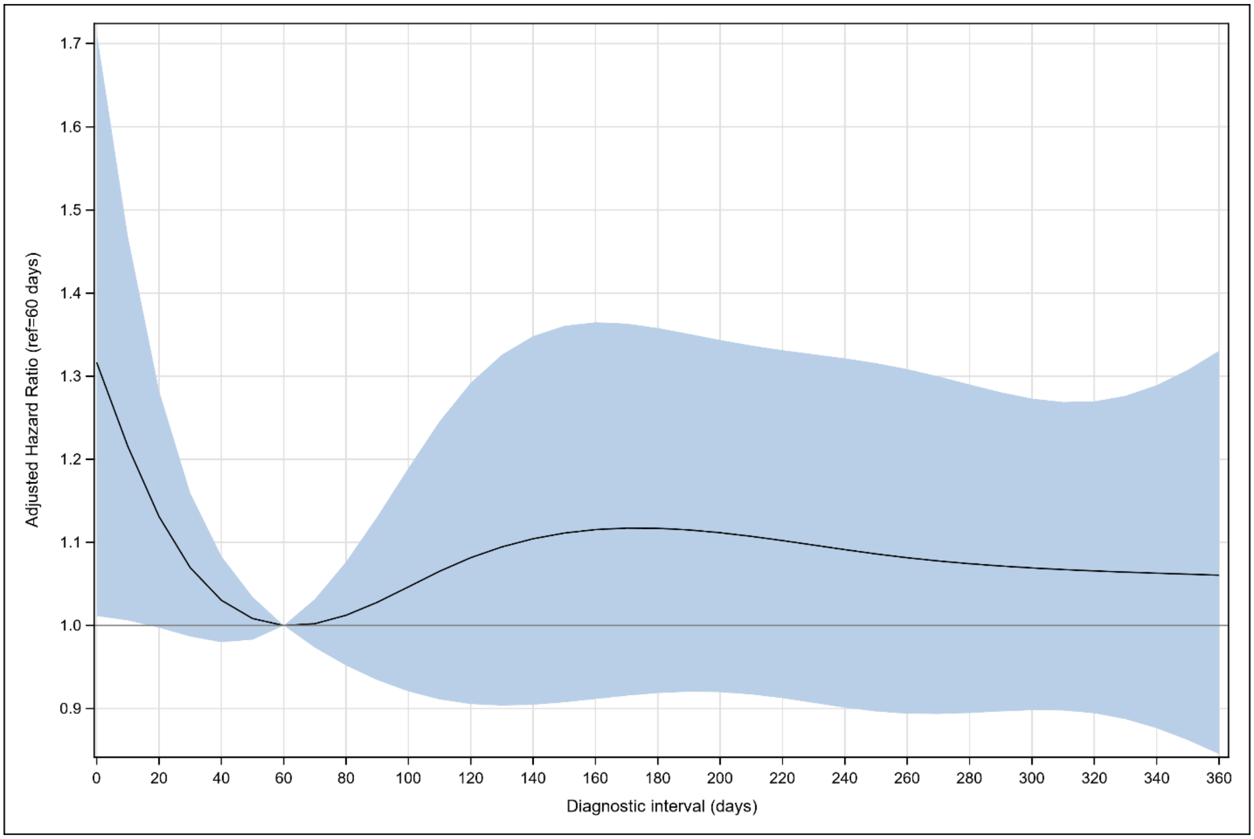

eFigure 1. Sensitivity Analysis Results Including Symptomatically Similar Diseases (SSDs) to Define the Start of the Diagnostic Interval

The relationship between diagnostic interval length and overall survival relative to a diagnostic interval length of 60 days, adjusted for covariates, with 95% confidence intervals.

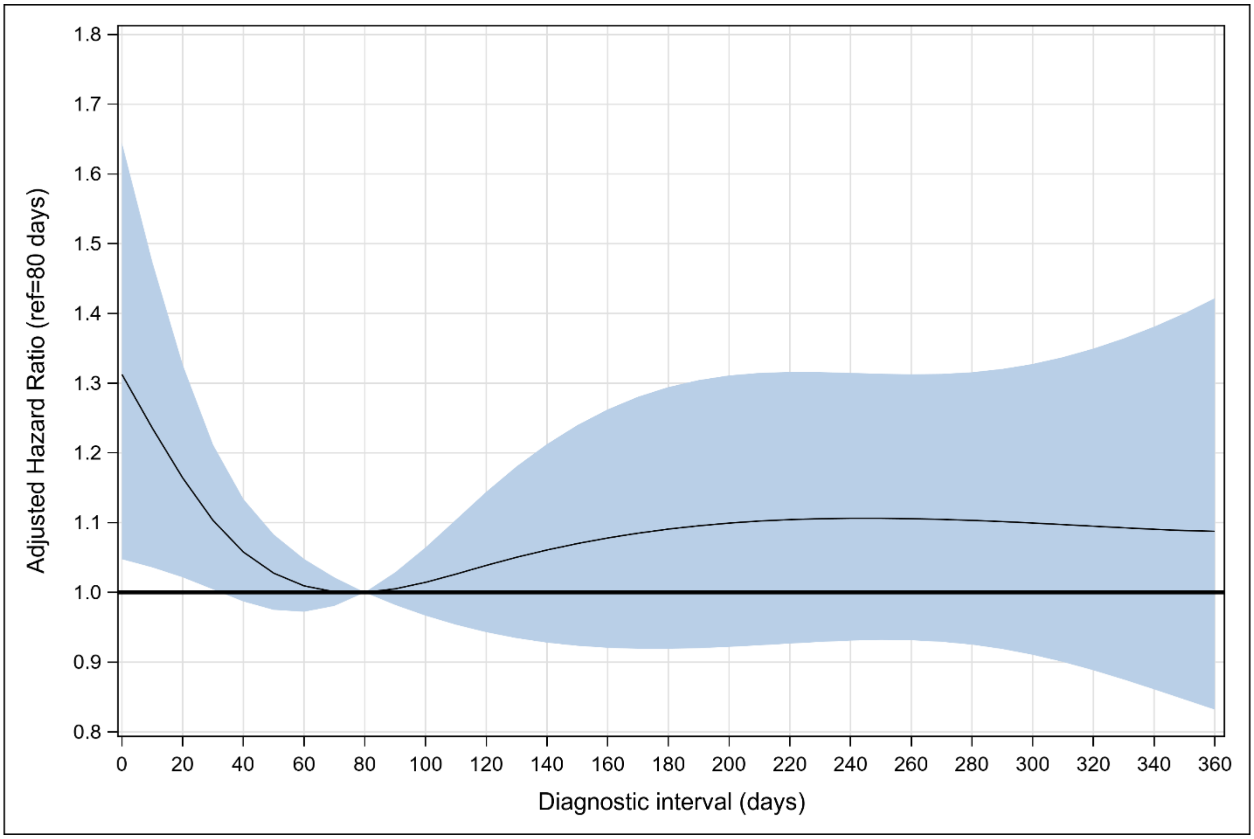

eFigure 2. Sensitivity Analysis Adjusting for Stage in the Association Between Diagnostic Interval Length and Survival

The relationship between diagnostic interval length and overall survival relative to a diagnostic interval length of 80 days, adjusted for covariates, with 95% confidence intervals.
