# Supplementary material for: Diagnostic Timing and Ovarian Cancer Survival
Source: JAMA Netw Open. 2026 Mar 27;9(3):e262434. doi: 10.1001/jamanetworkopen.2026.2434 (PMC13032158; doi:10.1001/jamanetworkopen.2026.2434)
Supplement: Supplement 2. — Data Sharing Statement [file jamanetwopen-e262434-s002.pdf]

Data Sharing Statement

Soppe. Diagnostic Timing and Ovarian Cancer Survival. *JAMA Netw Open*. Published March 27, 2026. doi:10.1001/jamanetworkopen.2026.2434

Data
Data available: No

Additional Information
Explanation for why data not available: Data includes potentially identifiable patient information and is protected by a DUA. Data can be accessed through an application to CIPHR at the UNC Lineberger Comprehensive Cancer Center.
